# Palmoplantar psoriasis: A clinicopathological correlation in a tertiary care hospital

**DOI:** 10.1111/srt.13882

**Published:** 2024-08-05

**Authors:** Gautam Das, Mahesh Mathur, Ayasha Shrestha, Sunil Jaiswal, Sailuja Maharjan

**Affiliations:** ^1^ Department of Dermatology College of Medical Sciences (COMS) Bharatpur Nepal; ^2^ Department of Dermatology Kulhudhuffushi Regional Hospital (KRH) Kulhudhuffushi Maldives; ^3^ Department of Community Medicine College of Medical Sciences (COMS) Bharatpur Nepal; ^4^ Department of Pathology Kulhudhuffushi Regional Hospital (KRH) Kulhudhuffushi Maldives

**Keywords:** clinicopathological, histopathology, palmoplantar psoriasis, psoriasis

## Abstract

**Background:**

Palmoplantar psoriasis is a clinical variant of psoriasis characterized by well‐defined erythematous desquamating plaques on palms and soles, which may or may not include pustules. Hyperkeratotic lesions of palm and sole commonly include Psoriasis, Eczema and Tinea. These conditions often present with overlapping clinical and histopathological features requiring clinicohistopathological correlation for a conclusive diagnosis. The presence of munro's microabscess or spongiform pustule of kogoj differentiates psoriasis of palm and sole from other hyperkeratotic lesions of palm and sole. The objective of this study was to study the clinical and histopathological profile of palmoplantar psoriasis and correlate clinical diagnosis with histopathological diagnosis.

**Method:**

A hospital‐based, descriptive study was conducted from January 1, 2020, to December 31, 2020. Fifty‐two patients were clinically diagnosed as palmoplantar psoriasis with or without involving other parts of body and routine histopathological evaluation was carried out as per standard protocols.

**Result:**

Clinically diagnosed 52 cases of palmoplantar psoriasis showed varied histopathology with hyperkeratosis (100%), parakeratosis (100%), regular acanthosis (75%), Supra‐papillary thinning (44.2%), spongiosis (65.4%), tortuous vessels in the papillary dermis (78.8%) and mixed inflammatory infiltrates (predominantly lymphocytic‐100%), which were observed to be prominent findings in skin biopsies of our patients. Clinicopathological correlation was achieved in 88.5% of cases.

**Conclusion:**

This study shows clinically diagnosed palmoplantar psoriasis with histopathological features consistent with palmoplantar psoriasis in 88.5% cases. Thus, clinically inconclusive hyperkeratotic lesions with palmoplantar psoriasis can be diagnosed with histopathological correlation improving the therapeutic intervention.

## 1 INTRODUCTION

Psoriasis is a chronic inflammatory autoimmune disease characterized by an excessive aberrant proliferation of keratinocytes.^1^ Palmoplantar psoriasis is a clinical variant of psoriasis affecting palms and soles.^2^

The skin lesion of psoriasis is characterized by well circumscribed erythematous, dry scaly plaques of varying sizes covered by silvery white scales having predilection for scalp, extensor surfaces of limbs, umbilical region, palms, soles and nails.^3^ There are various clinical variants of psoriasis like chronic plaque psoriasis, inverse psoriasis, generalized pustular psoriasis, erythro‐dermic psoriasis and palmoplantar psoriasis. The lesion of palmoplantar psoriasis is characterized by well‐defined erythematous desquamating plaques on palms and soles, which may or may not include pustules.^4^, ^5^, ^6^

Psoriasis affects approximately 2%–3% of the worldwide population of all age group regardless of ethnic origin.^7^, ^8^ Palmoplantar psoriasis constitute 3%–4% of all patients with psoriasis. Genetic and environmental factors play a role in the pathogenesis of psoriasis.^9^

Hyperkeratotic lesions of palm and soles commonly includes psoriasis, eczema and tinea. These lesions have certain common clinical presentation leading to confusion in diagnosis. Therefore, distinct histopathological features and clinical correlation will give a conclusive diagnosis. Typical histological features of palmoplantar psoriasis include hyperkeratosis, parakeratosis, acanthosis, elongation of rete ridges, suprapapillary thinning, munro's microabscess, spongiform pustule of kogoj, inflammatory infiltrates in the epidermis and dermis (neutrophils, mononuclear cells). The presence of munro's microabscess or spongiform pustule of kogoj differentiates psoriasis of palm and soles from other hyperkeratotic lesions of palm and soles.^10^, ^11^

Typical clinical and histopathological features are present in most of the cases of palmoplantar psoriasis. However, other hyperkeratotic lesions of palm and sole like hyperkeratotic eczema, tinea pedis/manum can have overlapping clinical features of psoriasis causing difficulty in diagnosis. Thus, distinct histopathological features and clinical correlation will give conclusive diagnosis.^10^, ^11^

The objective of our study was to study the clinical and histopathological profile of palmoplantar psoriasis and its correlation.

## 2 MATERIALS AND METHODS

A hospital‐based cross‐sectional descriptive study was conducted in all patients clinically diagnosed as palmoplantar psoriasis with or without involving other parts of body from January 1, 2020, to December 31, 2020. Patients diagnosed as palmoplantar psoriasis and already on systemic or topical medication were excluded from this study. The clinical diagnosis was based on detailed history and clinical examination. The presence of sharply demarcated and symmetrically distributed erythematous silvery scaly plaques with or without fissuring, studded pustules involving palm, knuckles of hand and soles, with presence or absence of similar type of lesion elsewhere in the body was taken clinically as a feature in favour of psoriasis.^12^ Unilateral hyperkeratotic scaly lesion suggestive of tinea manum or tinea pedis was excluded by 10% potassium hydroxide (KOH) examination. Biopsies were obtained from well‐developed lesion present on palm and sole and H&E (Hematoxylin and Eosin) stain was used for histopathological diagnosis.

Data was entered and analysed by Statistical Package for Social Sciences (SPSS) software (version 16). Data was analysed by using descriptive tool. In the descriptive statistics, bar‐diagram, mean and standard deviation were used. The level of significance in this study was considered as 5%.

## 3 RESULTS

Fifty‐two cases of clinically diagnosed palmoplantar psoriasis were included in our study and following observations were made, out of which, 46 (88.5%) were palmoplantar psoriasis and 6 (11.54%) were hyperkeratotic eczema.

### 3.1 Involvement of palm, sole and other site

**FIGURE 1:**
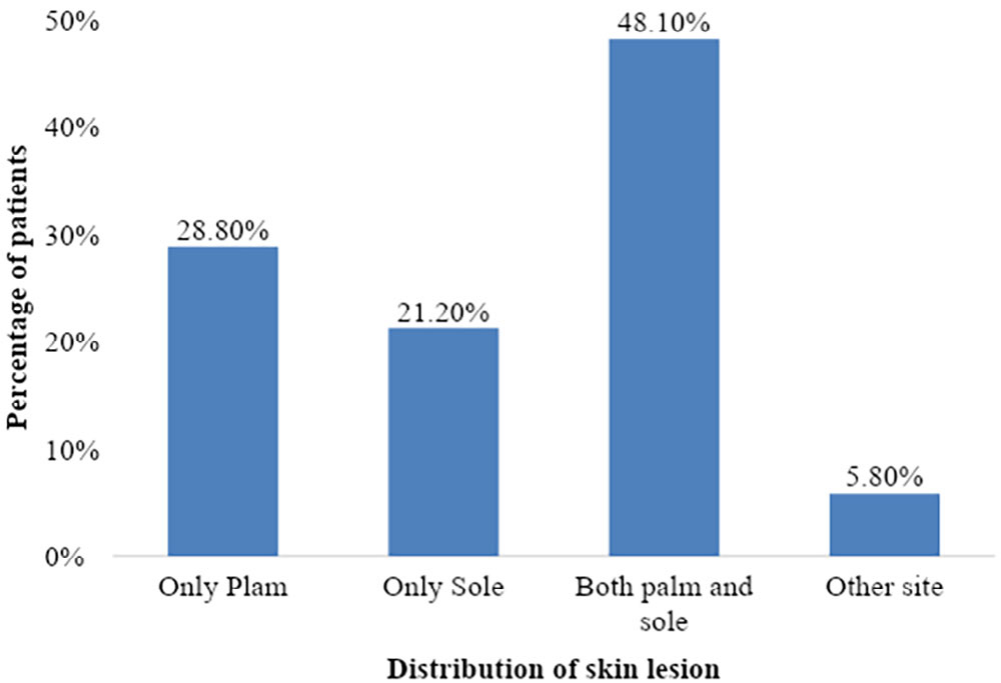
Bar diagram showing distribution of dermatoses on palm, sole and other site in our patients. (*n* = 52).

Among 52 cases, only palm was involved in 15 patients (28.8%, *n* = 15), only sole in 11 patients (21.2%, *n* = 11), both palm and sole in 25 patients (48.1%, *n* = 25) and three patients (5.8%, *n* = 3) had psoriasis on other sites, depicted in Figure 1.

### 3.2 Distribution of skin lesions in our patients

Symmetrical involvement was present in 51 patients (98%, *n* = 51), while only one case had asymmetrical involvement (2%, *n* = 1).

### 3.3 Distribution of lesion on palm and soles

**FIGURE 2:**
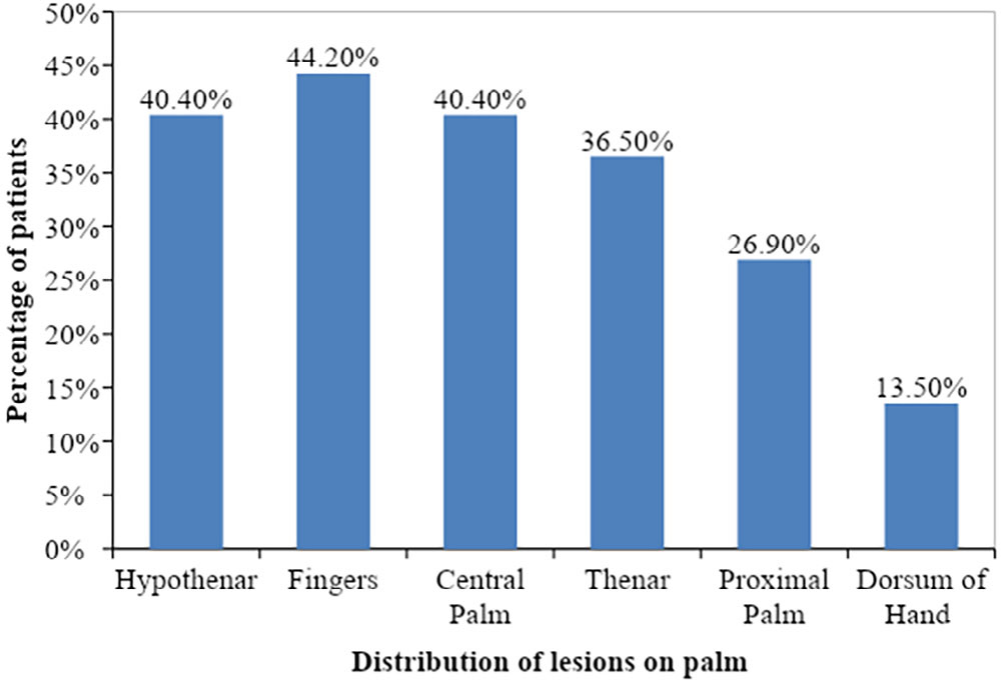
Bar diagram showing distribution of dermatoses over the palms in our patients (*n* = 52).

In palm, majority of patients had fingers involvement (44.2%, *n* = 23, Figure 5), followed by hypothenar (40.4%, *n* = 21, Figure 6), central palm (40.4%, *n* = 21), thenar (36.5%, *n* = 19), proximal palm (26.9%, *n* = 14), dorsum of hand (13.5%, *n* = 7) and whole palm (11.5%, *n* = 6) involvement as depicted in Figure 2.

**FIGURE 3:**
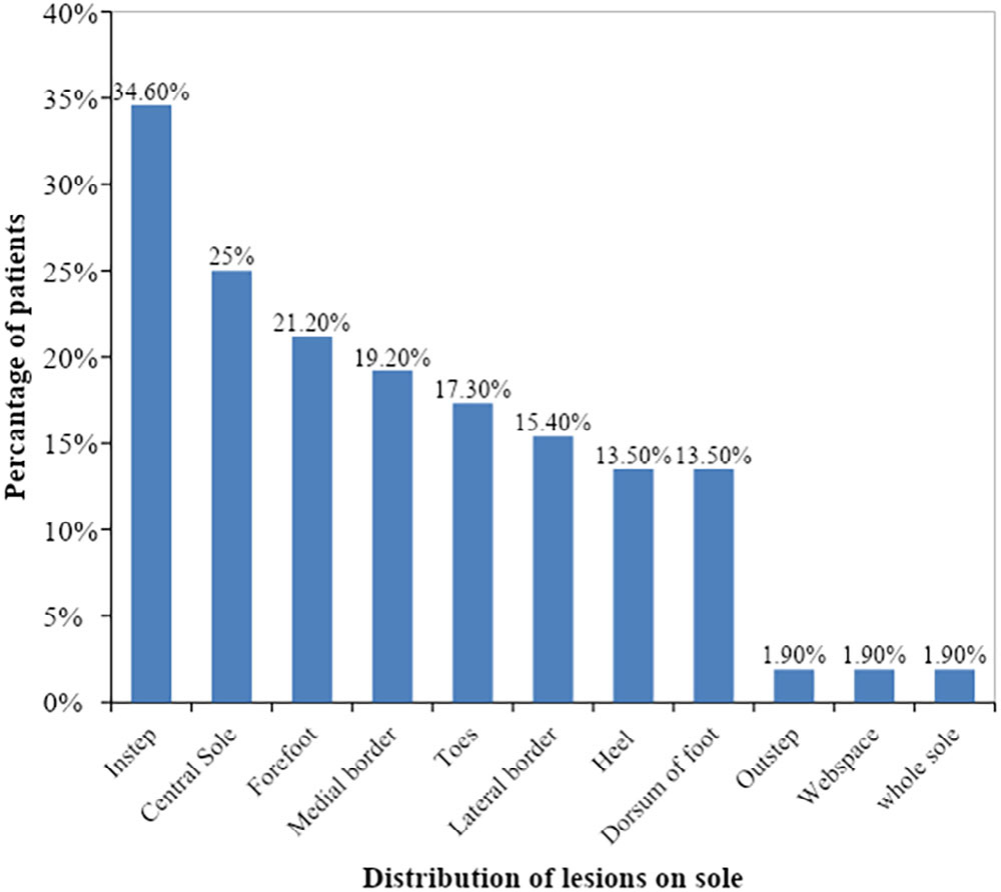
Bar diagram showing distribution of dermatoses over soles in our patients. (*n* = 52).

In sole, majority of patients had in‐step involved (34.6%, *n* = 18), followed by involvement of central sole (25%, *n* = 13, Figure 7), forefoot (21.2%, *n* = 11), medial border (19.2%, *n* = 10), toes (17.3%, *n* = 9), lateral border (15.4%, *n* = 8), heel and dorsum of foot (13.5%, *n* = 7), outstep, webspace, whole sole (1.9%, *n* = 1), depicted in Figure 3.

### 3.4 Nail changes

**FIGURE 4:**
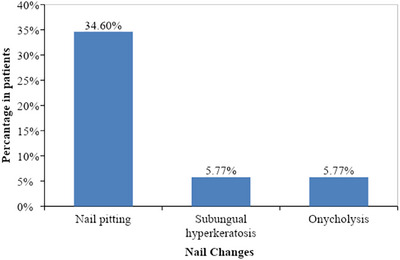
Bar diagram showing nail changes in our patients. (*n* = 24).

Nail changes were seen in 24 patients (46.1%, *n* = 24) with nail pitting being most common changes (34.6%, *n* = 18, Figure 8) followed by subungual hyperkeratosis (5.77%, *n* = 3, Figure 9) and onycholysis (5.77%, *n* = 3, Figure 10) as depicted in Figure 4.

**FIGURE 5:**
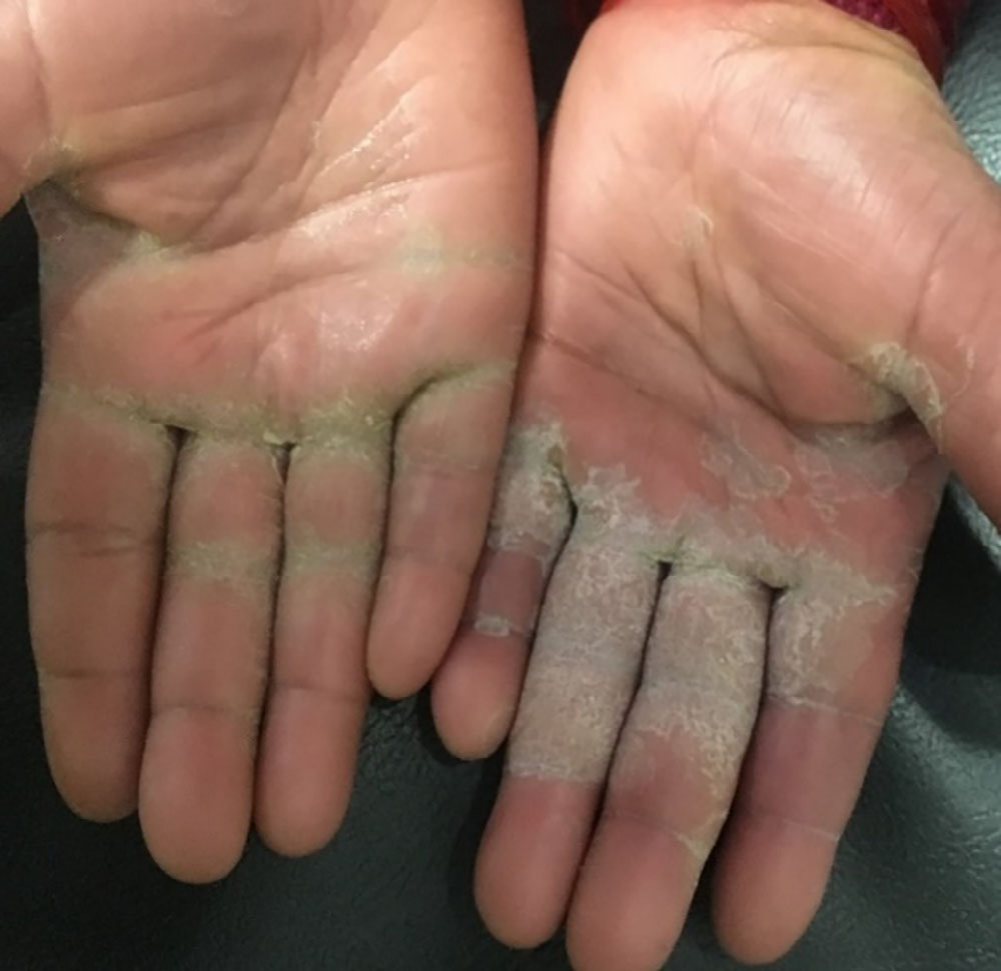
Psoriatic lesion involving the fingers of bilateral palm.

**FIGURE 6:**
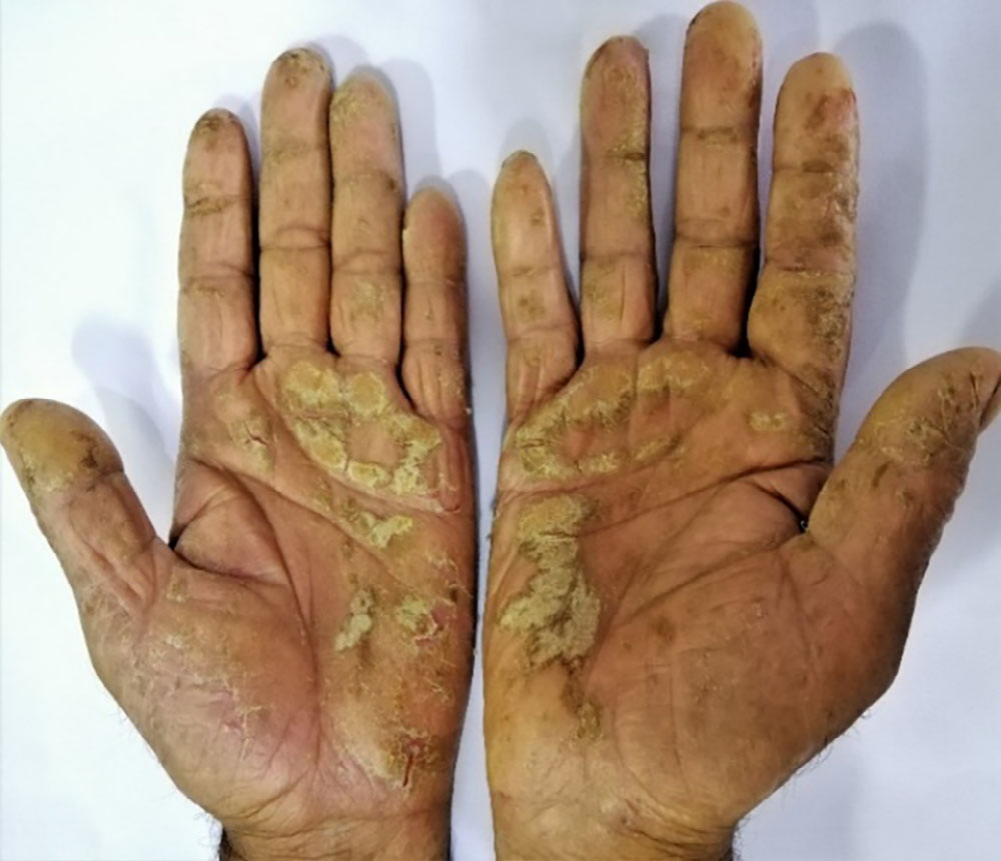
Symmetrical psoriatic lesion involving bilateral palm.

**FIGURE 7:**
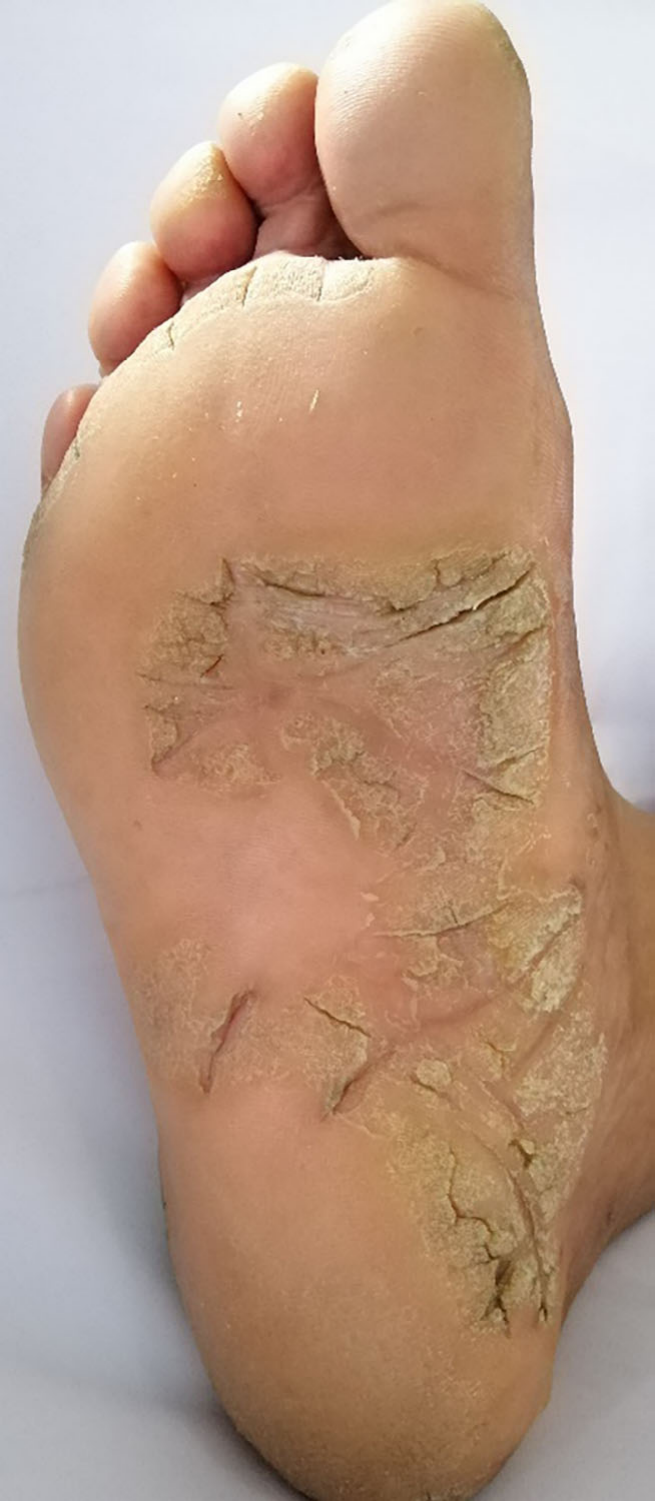
Psoriatic lesion involving inner central aspect of the foot on plantar aspect.

**FIGURE 8:**
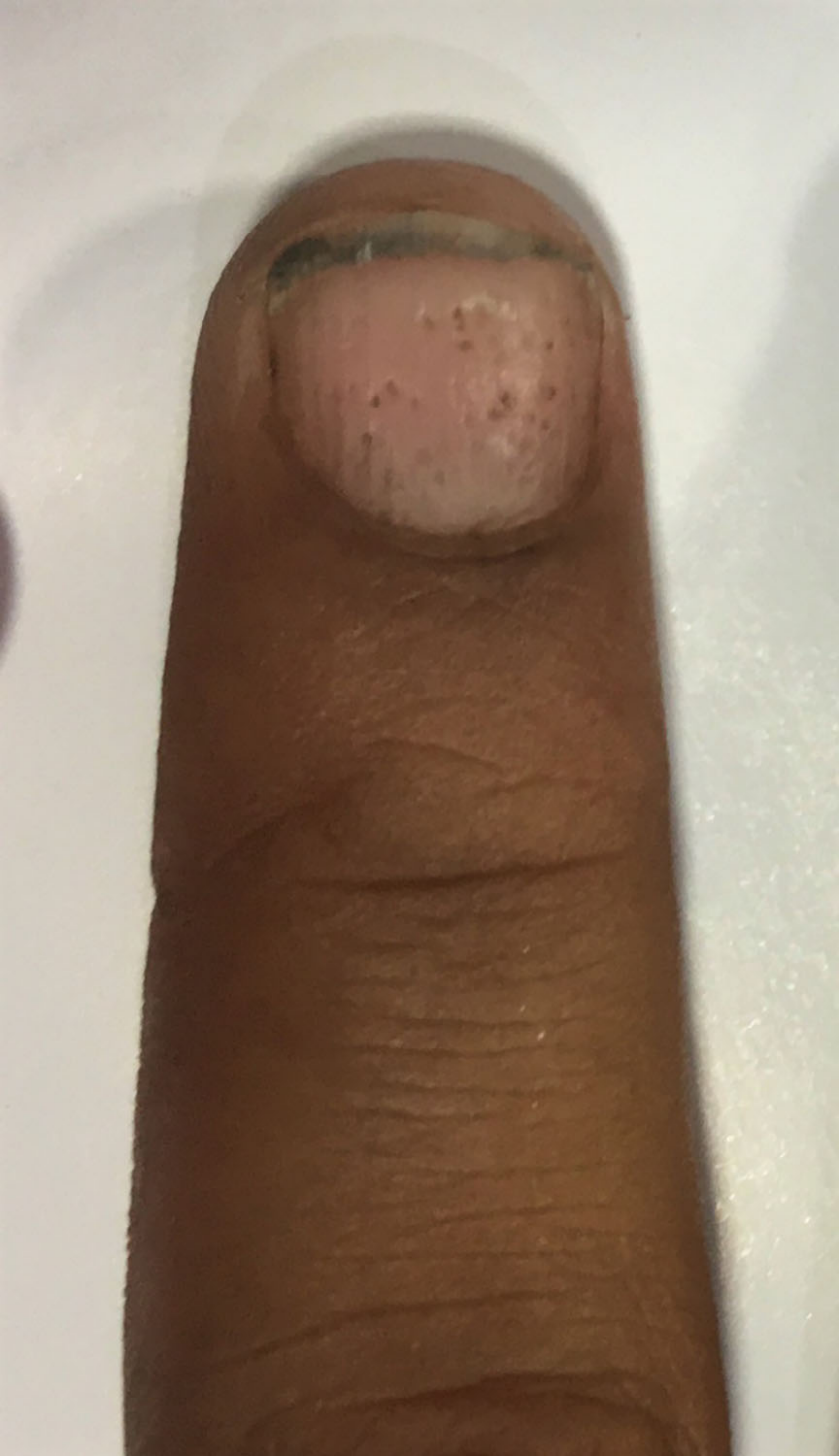
Nail pitting.

**FIGURE 9:**
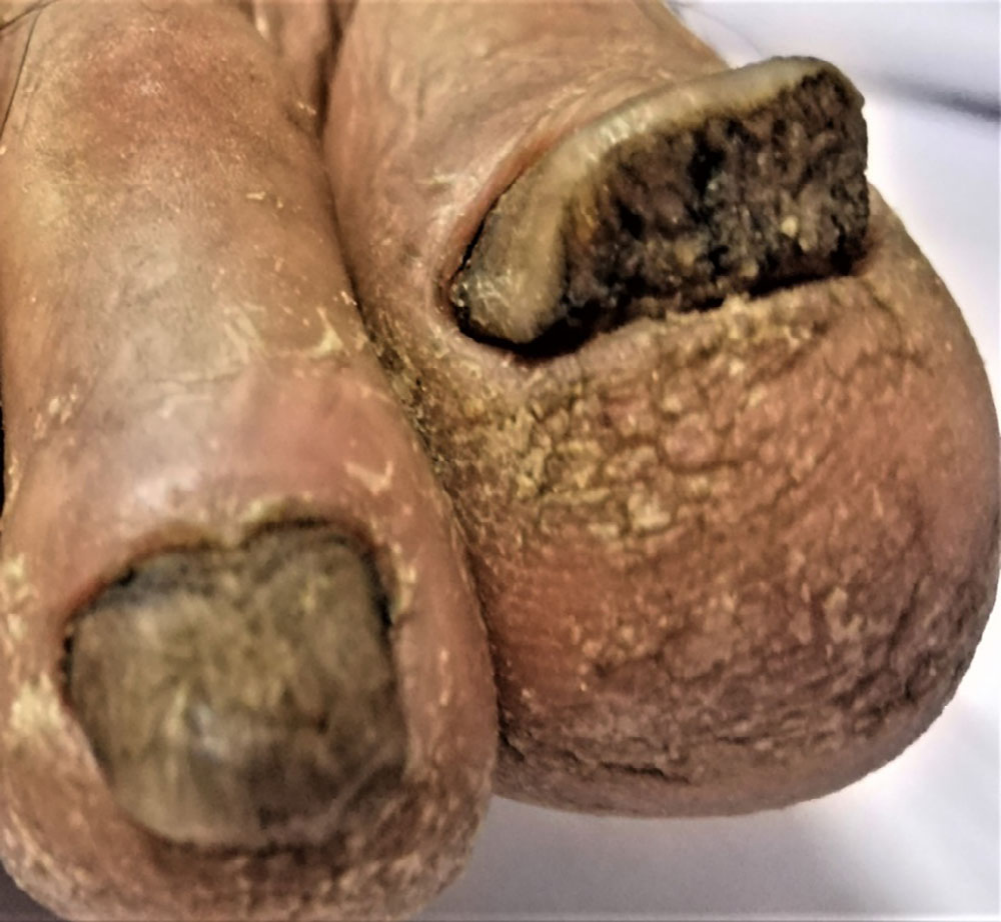
Subungual hyperkeratosis.

**FIGURE 10:**
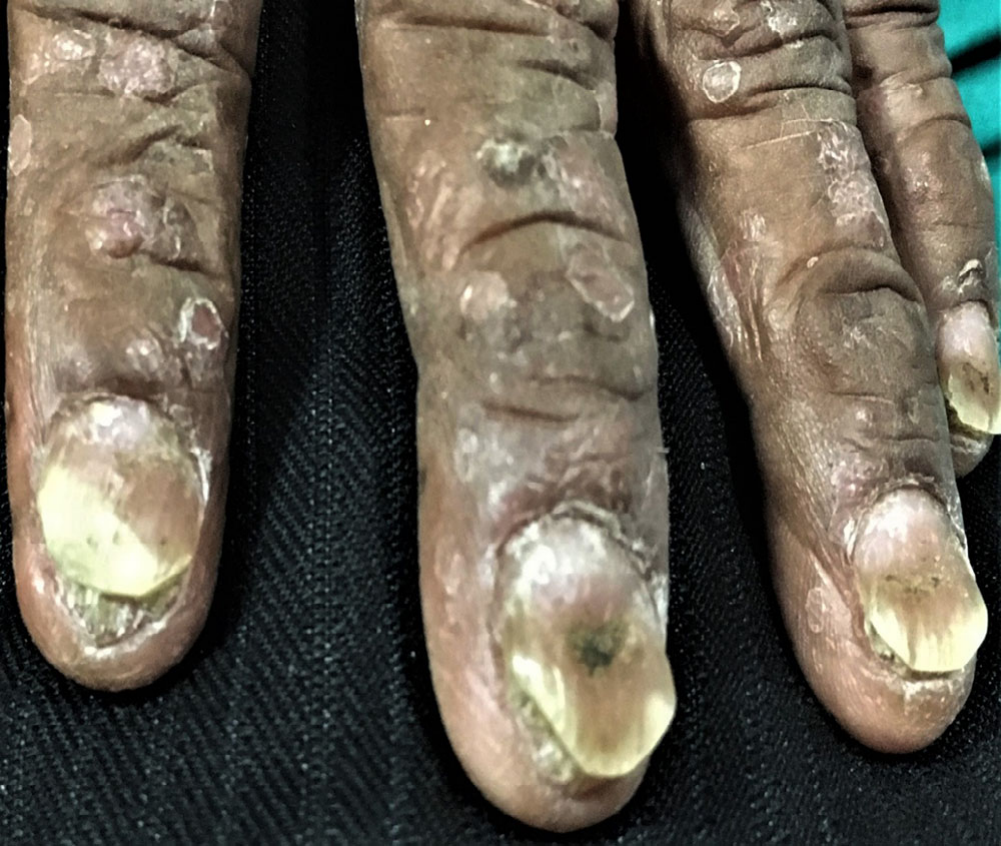
Distal onycholysis.

### 3.5 Histopathological data

Among 52 patients with clinical lesion suggestive of palmoplantar psoriasis, 46 patients had histopathological features consistent with clinical diagnosis (clinico‐pathological correlation of 88.5%, *n* = 46). Eczema was noted in six patients (11.5%, *n* = 6).

**FIGURE 11:**
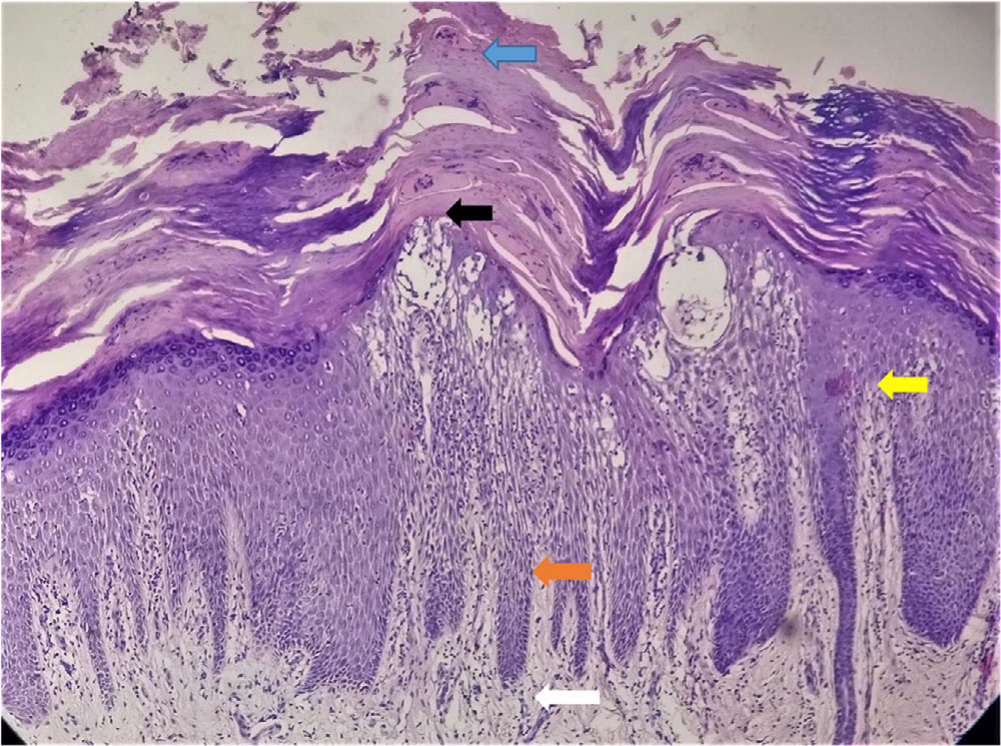
Histopathology of palmoplantar psoriasis showing hyperkeratosis with parakeratosis (blue arrow), focal hypogranulosis (black arrow), regular acanthosis (orange arrow), dilated tortous papillary dermal vessels (yellow arrow) and mild superficial dermal lymphocytic infiltration (white arrow). H&E stain, 10X.

**FIGURE 12:**
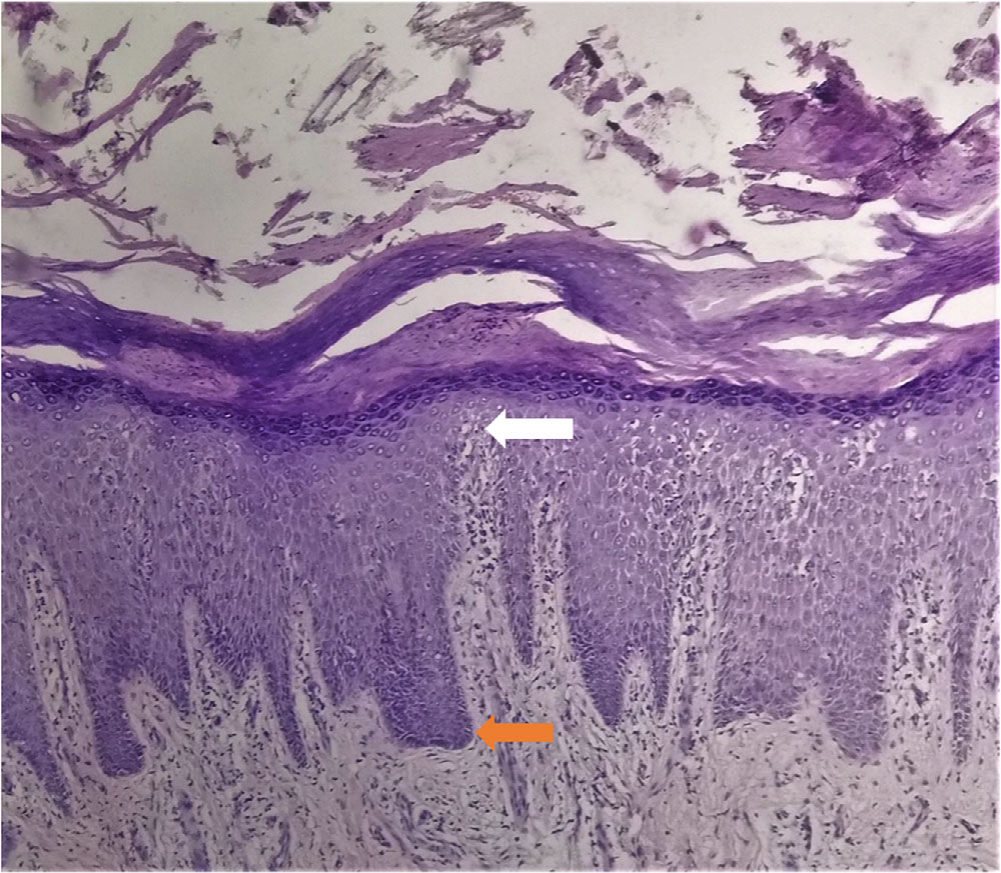
Suprapapillary thinning (white arrow) and regular elongation of rete ridges with characteristic clubbing of the tip (orange arrow). H&E stain, 10X.

**FIGURE 13:**
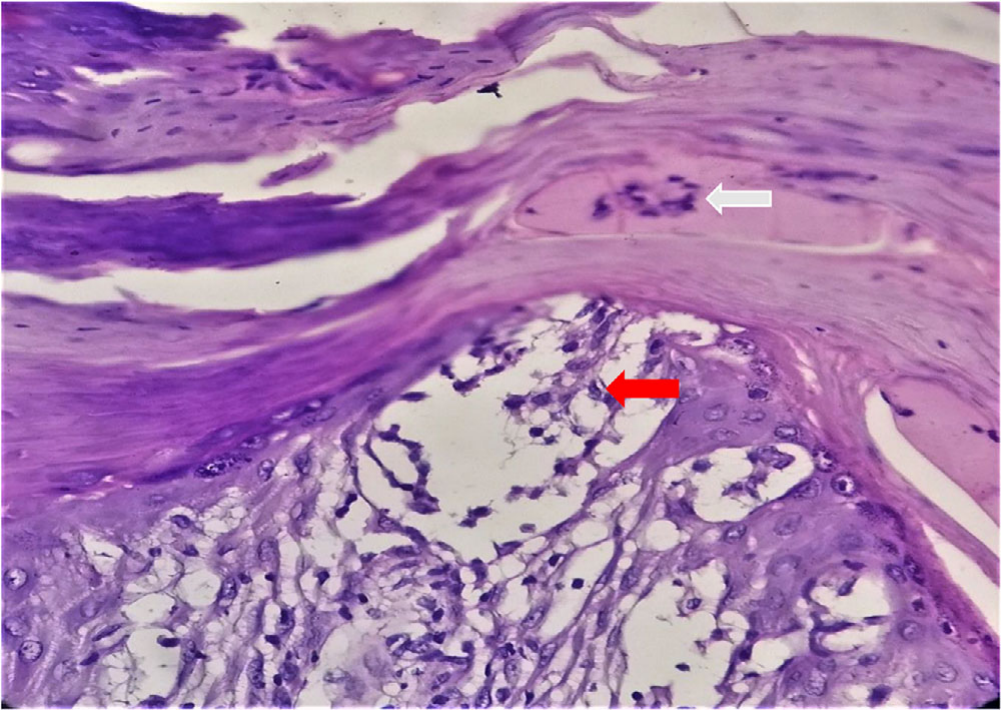
Munro's microabscess (white arrow) and spongiform pustules of kogoj (black arrow). H&E stain, 40X.

Histopathological findings observed in confirmed cases of palmoplantar psoriasis (*n* = 46) are summarized in Table 1 with significant findings depicted in Figures 11, 12, 13.

**TABLE 1:** Histopathological findings in palmoplantar psoriasis.

Histopathological findings	Present
Hyperkeratosis	100%
Parakeratosis	100%
Munro's microabscess	7.8%
Hypogranulosis	30.8%
Acanthosis	25% (Irregular)
	75% (Regular)
Spongiform pustules of kogoj	1.9%
Spongiosis	65.4%
Suprapapillary thinning	44.2%
Elongation of rete ridges	63.5%
Tortous vessels in papillary dermis	78.8%
Dermal oedema	3.9%
Inflammatory infiltrates (predominantly lymphohistiocytic)	100%

Overall, a clinico‐pathological correlation was obtained in 88.5% cases (46 out of 52) as depicted in Table 2.

**TABLE 2:** Clinico‐pathological correlation in a clinically diagnosed palmoplantar psoriasis patients.

Clinical diagnosis	Histopathological diagnosis	Clinico‐pathological Correlation
Palmoplantar psoriasis (100%, *n* = 52)	Palmoplantar psoriasis (88.5%, *n* = 46)	(88.5%, *n* = 46)
	Hyperkeratotic eczema (11.5%, *n* = 6)	

## 4 DISCUSSION

Palmoplantar psoriasis is a subtype of psoriasis with a prevalence of 3% in general population.^9^ Palmoplantar psoriasis often presents with similar and overlapping clinical and histopathological features with eczema, tinea manum/pedis and palmoplantar keratoderma of palm and soles and leads to diagnostic dilemmas, which has direct impact on therapeutic intervention. The recurrent nature and prognosis of psoriasis differs from eczema, tinea, palmoplantar keratoderma of palm and sole. Thus, clinical and histopathological study will give conclusive diagnosis. This study was undertaken to evaluate the clinical and histopathological characteristics along with clinico‐histopathological correlation of palmoplantar psoriasis.

Psoriasis usually affects all age group regardless of ethnic origin but most commonly affects individuals during their second to fourth decade of life.^8^, ^13^

Psoriasis of palms and soles present as erythematous sharply circumscribed plaques with silvery white scales and peripheral overhanging scale on the palms, sides of fingers, finger tips and on extensor surfaces of joints.^14^ Palmoplantar psoriasis is shown to constitute 3%−4% of all psoriasis cases in most of the studies conducted.^15^ In our study, majority of our patients (48.1%) had both palm and sole involvement followed by involvement of only palm in 28.8%, only sole in 21.2% and psoriasis on other sites in 5.8% as depicted in Figure 1. This was in accordance with study conducted by Khandpur et al.^16^ in 2011 having both palm and sole involvement in 48%. However, study conducted by Nair et al.,^17^ in 2017, and Suman Babu et al.,^15^ and kamyab‐Hesari et al.,^18^ in 2014, showed exclusive palmar involvement in 25.74% and 20%, respectively. Both palm and sole involvement in our study can be explained due to the fact most of our patients were housewives and farmer involved in manual work in farm that may point to the role of Koebner's phenomenon.

In a study by Agarwal et al.,^11^ in 2014, and Khandpur et al.,^16^ in 2011, reported 80% and 79% cases, respectively, having symmetrical psoriatic lesions in palmoplantar psoriasis, which was comparable to our study having symmetrically distributed lesion in 98% cases.

Khandpur et al.,^16^ in their study of palmoplantar psoriasis, found pressure areas commonly involved with 44% fingers, 11% thenar and 15% hyothenar eminences, with centre and distal aspects of palms being less frequently affected. This was in accordance to our study having fingers (44.2%) most commonly involved sites in the palm, followed by hypothenar and central palm (40.4%), thenar (36.5%), proximal palm (26.9%), dorsum of hand (13.5%) and whole palm (11.5%) as depicted in Figure 2. In our study, fingers were most commonly involved, which might be explained by the fact that Koebner's phenomenon occurs at friction and traumatic sites in fingers while working in our patients (especially in housewives and farmer).

In our study, in soles, instep (34.6%), central sole (25%), forefoot (21.2%), sides of feet, heel were commonly affected, with outstep, web space and whole sole less commonly affected sites as depicted in Figure 3, which was comparable to study conducted by Khandpur et al.,^16^ in 2011, reporting instep commonly involved sites in sole (37.6%). Web space involvement reported by Khandpur et al.,^16^ in 2011, was 28.57%, which was much high than we observed in our study in only 1.9% of cases. No obvious reason was observed.

Nail involvement is common in psoriasis and might be the first manifestation of psoriasis and may only be the site of involvement in some patients reported.^19^ About 80%−90% of the psoriatic patients will develop nail psoriasis at some point of time during the course of the disease. The effects of psoriasis in the nail matrix, nail bed leads to changes in nail.^20^

In our study, nail changes were seen in 40.3% cases with nail pitting being the most common nail changes present in 34.6% of cases, followed by 5.77% onycholysis, and subungual hyperkeratosis as depicted in Figure 4. Our results are consistent with studies carried out by Suman Babu et al.,^15^ having nail pitting most common nail changes followed by subungual hyperkeratosis (36%).

Histopathological features most useful for diagnosis of psoriasis are Munro's microabscess (collection of neutrophils within parakeratosis), spongiform pustule of kogoj (neutrophils within the spinous layer), dilation of papillary dermal capillaries with overlying thinning of the suprapapillary epidermis. Palmoplantar psoriasis can be differentiated from palmoplantar eczema and Tinea pedis/manum by the presence of munro's microabscess, spongiform pustule of kogoj and suprapapillary thinning in histology.^21^

Histopathologically, in our study, hyperkeratosis and parakeratosis were present in skin lesions of all our patients, which was in accordance to Agarwal et al.,^11^ in 2014, having hyperkeratosis (75.67%), parakeratosis (97.3%), Kamyab‐Hesari et al.,^18^ in 2014, having parakeratosis (100%) and Rao et al.,^12^ in 2018, having parakeratosis (90.4%) cases.

A decrease or absence of granular layer in the suprapapillary area is an important finding in psoriasis.12 A study by Rao et al.,^12^ in 2018, reported hypogranulosis in 22.6% cases, which was comparable to our study having hypogranulosis in 30.8% cases. However, other similar studies by Park et al.,^22^ in 2017, and Kamyab‐Heasari et al.,^18^ in 2014, reported hypogranulosis in 62.5% and 75%, respectively, which was much higher compared to our study (30.8%). There is profound variable of hypogranulosis in reported literature and no specific reason was observed.

Several previous studies have reported regular acanthosis to be more common than irregular acanthosis in palmoplantar psoriasis.^22^, ^23^ This was in accordance with our study having regular acanthosis (75%) and irregular acanthosis (25%).

Studies by Agarwal et al.,^11^ in 2014, and Park JY et al.,^22^ in 2017, reported elongation of rete ridges in 100% and 87.5% cases, respectively. This was higher than the result (63.5%) reported in our study. No obvious reason was observed.

Spongiosis, mostly in the lower half of the epidermis, has been observed to be a prominent finding in palmo‐plantar psoriasis.^24^ In our study, spongiosis was detected in 65.4%, while other similar studies reported variable results from 60% to 82%.^11^, ^12^, ^25^, ^26^

Two key histological markers of psoriasis are the findings of foci of neutrophils in the parakeratotic stratum corneum, known as “Munro's microabscess”, and spongiform neutrophilic micropustules in the spinous layer of the epidermis, termed “spongiform pustules of Kogoj”.^27^ In our study, Munro's microabscess was present in 7.8% of cases and spongiform pustules of kogoj in 1.9%. These findings were variable in other reported studies. ^11^, ^12^, ^25^

Studies by kamyab‐Hesari et al.,^25^ in 2014, and Agarwal P et al.,^11^ in 2014, reported suprapapillary thinning in 72.2% cases and 94.59%, respectively, to be a strong pointer towards the diagnosis of psoriasis. However, no specific reason was reported. In our study, suprapapillary thinning was present in 44.2% cases, which was in agreement with the study reported by Rao et al.,^12^ in 2018, having suprapapillary thinning in 51.7% cases.

In a study by Kamyab‐Hesari et al.,^18^ in 2014, who demonstrated dilated vessels in 64% of palmoplantar psoriasis considered this finding to be a strong pointer towards psoriasis, which was consistent with our study and Park et al.,^22^ in 2017, having dilated tortuous vessels in 78.8% and 97.5% cases.

The nature of dermal inflammation was predominantly lymphocytic (100%) in all our patients along with neutrophils in few cases. This was consistent with study by kamyab‐Hesari et al.,^18^ who reported mixed inflammatory infiltrates (consisting predominantly lymphocytes: 97.2%, neutrophils: 38.9% and eosinophils: 38.9%). Similarly, other studies by Rao et al.^12^ and Agarwal et al.^11^ also reported mixed inflammatory infiltrates. Thus, psoriasis is T‐cell mediated inflammation.

In our study, dermal oedema was seen in 3.9% of cases as reported by Rao et al.,^12^ in 2018, and Kamayab‐Hesari et al.,^18^ in 2014 having 19% and 83.3% cases, respectively, while studies reported by Agarwal et al.^11^ and Park et al.^22^ did not show dermal oedema. Thus, the dermal oedema in histopathological findings in palmoplantar psoriasis was variable with inconsistent finding.^11^, ^12^, ^18^, ^22^

In a study by Pandit et al.,^27^ in 2015, and Mehta et al.,^28^ in 2009, reported clinico‐histopathological correlation in psoriasis to be 95.24% and 72.4%, respectively. In our study, clinico‐pathological correlation was observed in 88.5% cases (46 out of 52 patients) as depicted in Table 2. 11.5% (6 out of 52 patients) patients were histologically diagnosed as hyperkeratotic eczema on the basis of moderate to severe spongiosis with absence suprapapillary thinning of epidermis, tortuous vessels in papillary dermis, munro's microabscess and spongiform pustule of kogoj.

## Data Availability

The data that support the findings of this study are available from the corresponding author upon reasonable request.
